# Prevalence and Predictors of Self-Medication with Antibiotics in Al Wazarat Health Center, Riyadh City, KSA

**DOI:** 10.1155/2016/3916874

**Published:** 2016-01-05

**Authors:** Abdulrahman Al Rasheed, Umar Yagoub, Hesham Alkhashan, Osama Abdelhay, Ahmad Alawwad, Aboud Al Aboud, Saad Al Battal

**Affiliations:** ^1^Department of Family and Community Medicine, Prince Sultan Military Medical City, P.O. Box 7897, Riyadh 11159, Saudi Arabia; ^2^Research Unit, Family and Community Medicine Department, Prince Sultan Military Medical City, P.O. Box 7897, Riyadh 11159, Saudi Arabia; ^3^Al Wazarat Health Center, Prince Sultan Military Medical City, P.O. Box 7897, Riyadh 11159, Saudi Arabia; ^4^Training and Research Unit of Family and Community Medicine Department, Prince Sultan Military Medical City, P.O. Box 7897, Riyadh 11159, Saudi Arabia

## Abstract

*Background.* Antibiotics are responsible for most dramatic improvement in medical therapy in history. These medications contributed significantly to the decreasing mortality and morbidity when prescribed based on evidence of microbial infection.* Objective.* The aim of this study was to determine the prevalence and predictors of self-prescription with antibiotics in Al Wazarat Health Center, Riyadh City, Kingdom of Saudi Arabia.* Material and Methods.* Cross-sectional study was conducted in Al Wazarat Health Center between February 2014 and November 2014. Respondents were randomly selected using a multistage clustered random sampling technique. Data was entered into SPSS version 21 and analyzed. Descriptive statistics and multiple logistic regression models were applied.* Results.* A total of 681 patients have participated in this study with a response rate of 92%. The prevalence of self-prescription with antibiotics in Al Wazarat Health Center was 78.7%. Amoxicillin was the most used self-prescribed antibiotic with prevalence of (22.3%). Friend advice on self-prescription of antibiotics use (*p* = 0.000) and pharmacy near to the participants (*p* = 0.002) were the most common predictors for self-prescription with antibiotics.* Conclusion.* The level of self-prescribing antibiotics is relatively high among participants. Health education on the appropriate use of antibiotics is highly recommended. The proper use of treatment guidelines for antibiotic therapy will significantly reduce self-prescription with antibiotics.

## 1. Introduction

In recent years, the use of nonprescribed antibiotics had become a major global public health problem [1, 2]. It is estimated that more than 50% of antibiotics worldwide are purchased privately without a prescription, from pharmacies or street vendors in the informal sector [3]. This indiscriminate use of antibiotics contributes towards the spread of antimicrobial resistance globally [4, 5]. Resistance of the microorganisms to treatment will definitely lead to treatment failure and endangering patients' life [6, 7]. Microbial infections are most common types of infection which usually requires the use of antibiotics for treatment [8, 9]. Nondoctor prescription of antibiotics has severe adverse health effects and economically billions of Saudi Riyals are spent yearly for shifting patients to the second line of antibiotics as results of the failure of the first line of treatment [10].

Saudi Arabia is one of the richest developing countries in the Middle East with a fast growing population. Saudi Arabia has been described as a country with worldwide significance in the context of global epidemiology of antimicrobial resistance [11]. The country has an excellent health system which takes care of the health of its citizens both civilian and military. Currently the health authorities are faced by several health challenges such as self-prescription of antibiotics which could lead to the development of microbial resistance, cross-resistance, and treatment failure. This issue affects all population including the military personnel. As it is known military personnel are the backbone of any country, shouldering the defense of its nation from foreign invaders and enforcing low and order.

In the developing world, the unsuccessfully treated bacterial infection is rising on daily basis and more human lives are lost [12, 13]. Most studies have shown that self-prescription and nondoctor prescription may be due to the high cost of antibiotics, high cost of doctor consultation, and other economic factors [14–16]. On the other hand Saudi Arabia, a rich country with mostly free medications and free doctor consultations in most governmental hospitals, however still shows high self-prescription and nondoctor prescription. There were no many published studies on self-prescribed antibiotics in Riyadh City. This study will not only determine the prevalence rate of self-prescription of antibiotics but also explore the reasons for self-prescription and identify the illnesses necessitated by self-prescription among military personnel. The main justification for conducting this study was the fact that self-prescription with antibiotics is associated with development of resistance, cross-resistance, and treatment failure.

## 2. Material and Methods

### 2.1. Study Design, Sample Size Calculation, and Sampling

This study is a hospital based cross-sectional study. It used structured questions to assess nonprescribed antibiotic usage among military and nonmilitary personnel who attended Al Wazarat Health Center in Prince Sultan Military Hospital in Riyadh, Saudi Arabia. Al Wazarat Health Center is one of the primary healthcare centers under the Medical Service Department (MSD) at the Ministry of Defense in Saudi Arabia. The center consists of 20 specialized clinics which provide services on daily basis. It is accredited by Joint Commission International Accreditation (JCIA). The MSD is one of the main healthcare providers in the country along with the Ministry of Health and the private sector.

The study population was adults (over 18 years old) living in Riyadh City, with no disabilities that would prevent understanding the questionnaire. Respondents were randomly selected using a two-stage cluster sampling method. First-stage sampling included selection of six health clinics, namely, Chronic Disease Clinic, Dermatology Clinic, Flu clinic, ENT Clinic, Nephrology Clinic and Neuro-Spinal Clinic, from the total of 20 clinics providing services in Al Wazarat Health Center. The second stage of sampling was selecting 126 patients from each of these six clinics using simple random sampling method among the participants who satisfied the inclusion criteria to achieve the required sample size; the questionnaire was then administered to each of the participants (Table 1).

The sample size required to study the self-medication with antibiotics in Riyadh City was calculated to be 757 subjects based on the following formula:

(1) $$\begin{array}{c} n=\frac{3.84\vartheta^{2}}{d^{2}}, \end{array}$$

where *ϑ* ^2^ = (0.685)^2^ is the estimated standard deviation of a pilot test and *d* ^2^ = (0.05)^2^ is the desired precision. Therefore, the sample size required was calculated to be 721 respondents. We allowed 5% for incomplete data or missing values in the data, and the required sample size was increased to be 757 participants.

### 2.2. Study Instrument and Data Collection

A questionnaire was developed based on literature in which self-medication with antibiotics was examined in Asian countries. A combination of open-ended and close-ended questions was used. The questionnaire consisted of three parts' sociodemographic characteristics such as age, gender, marital status, occupation and educational level, reasons for self-prescription of antibiotics, and symptoms or illness for which antibiotics are self-prescribed (coughs, common cold, sore throat, fever, pneumonia, stomach ache, diarrhea, urinary tract infection, unintentional injury, skin disease, and no symptoms). Two independent bilingual translators were selected to make a forward and backward translation from English language into Arabic language. The translators were asked to make a semantic translation and the study instrument was then checked for accuracy of translation. The questionnaire was assessed for face and content validity by a group of local experts, that is, three pharmacists, two medical doctors and a psychologist. This consultation process led to redrafting and reorganizing items in the questionnaire. The questionnaire was pilot tested on 15 people, who were representative of the study population, to determine the clarity of the language and questionnaire structure. Some words were changed based on responses. The instrument underwent test retest and then was validated before it was finally administered to study participants.

Data collection was conducted over 3 months from May to July, 2014, by two trained research assistants. The questionnaires were self-administered to participants in a private room after participants willingly and voluntarily agreed to participate in the study.

### 2.3. Data Analysis and Modeling

Data were entered, analyzed, and digitally stored with the assistance of statistical descriptive statistics that were used to describe sociodemographic characteristics of the respondents, the point prevalence, and the patterns of self-medication with antibiotics Statistical Package for the Social Sciences (SPSS) version 22 [17]. A cross-tabulation analyses with odds ratio test were performed to identify the association between the demographic characteristics of the respondents and the status of self-prescription of antibiotics. Multiple logistic regression analysis was carried out to determine the predictors of self-prescription of antibiotics. Three different logistic regression techniques were used—the enter method, forward likelihood ratio, and backward likelihood ratio. Interaction was carefully examined and likely interaction terms were tested before the final model was produced.

The predictor variables were the sociodemographic characteristics (gender, marital status, education, work status, age, and job type), reasons for self-prescription of antibiotics (advice from friend or relative, lack of time, high consultation fees, hospital or clinic being too far, ease of obtaining drugs, convenience, past experience, and mild illness), and symptoms or illness for which antibiotics are self-prescribed (coughs, common cold, sore throat, fever, pneumonia, stomach ache, diarrhea, urinary tract infection, unintentional injury, skin disease, and no symptoms).

To predict factors affecting self-prescription with antibiotics we used a multiple logistic regression model. The following formula is to predict the probability of being self-prescribed with antibiotics given some selected variables. *Z* = −4.163 + 1.999 (Friend advice) + 1.277 (Pharmacy near) + 1.070 (Age group iii) − 1.104 (Job type) − 0.814 (University) was used. The legit coefficient was −1.735 and the odds ratio was 0.176 [Exp(−1.735)1 = 0.176].

### 2.4. Ethical Approval

Ethics approval was obtained from the ethical committee at Military Service Department at Ministry of Defense and research ethics committee at research center in Prince Sultan Military Medical Center under project number 622. All of the participants verbally consented and agreed willingly and voluntarily to participate in the study.

## 3. Results

### 3.1. Demographic Factors and Self-Prescription

Among the 757 distributed questionnaires 59 patients refused to participate in the study and 17 questionnaires were incomplete and removed from the analysis. A total of 681 participants had correctly filled the questionnaires; the response rate was 92%.  Table 2 shows the cross-tabulation results of sociodemographic variables and the status of self-prescription of antibiotics. The relationship of six demographic factors (gender, marital status, education, work status, age, and job type) with status of self-prescription of antibiotics was examined. Of the demographic factors, only the following three variables “gender, age, and job type” were significantly associated with the status of self-prescription of antibiotics.

Demographics of study participants are summarized in Table 2. Study participants were more likely to be males who were 523 (76.8%) while the remaining 158 (23.2%) were females. Male participants have higher proportion of self-prescribed antibiotics compared to their female counterparts, according to the odds of being self-prescribed antibiotic user among men that was 10.28 (95% confidence interval [CI]: 6.771, 15.601) times higher than the reference group of the female respondent group.

Older respondents were more likely to be self-prescribed antibiotic users than younger respondents. The probability of self-prescription of antibiotics among the age group of 31–44 years was almost double [odds ratio: 1.878 (95% CI: 1.223, 2.882)] that of the reference age group of 18–30 years; while the probability of self-prescription of antibiotics among the age group of ≥45 years was four times [odds ratio: 3.655 (95% CI: 2.195, 6.084)] more than that of the reference age group of 18–30 years. Finally, with regard to the job type, majority of the respondents were civilian, 458 (67.3%), where military respondents were 223 (32.7%); in addition, military respondents were less likely to be self-prescribed antibiotics users as they have 84.2% chances of being self-prescribers of antibiotics [odds ratio: 0.158 (95% CI: 0.106, 0.236)]. Other sociodemographic characteristics such as gender, marital status, education, and work status were not statistically significant related to the status of self-prescription of antibiotics.

**Figure 1:**
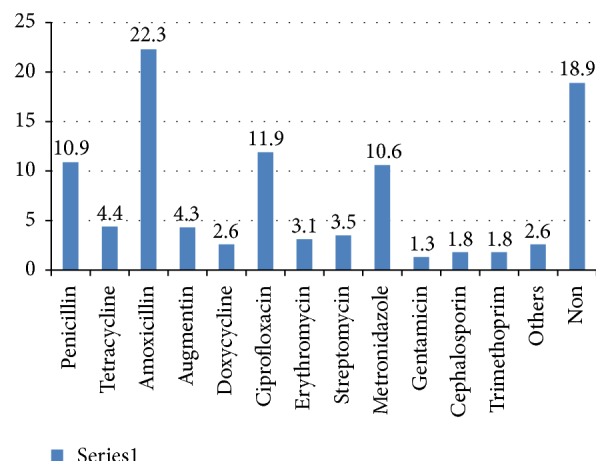
Prevalence of self-prescribed antibiotics (*n* = 536).

Of the total 681 observed encounters, 536 (78.7%) were involved with self-prescription of antibiotics, while 145 (21.3%) have not been involved with self-prescription of antibiotics in the last six months.  Figure 1 lists 12 different antibiotics that are most commonly used by the study participants. The vast majority of nonprescribed antibiotics are systemic antibiotics such as Amoxicillin, Ciprofloxacin, and Penicillin. It is reported that Amoxicillin is the most used self-prescribed antibiotic of the study respondents claiming (22.3%) participants, Ciprofloxacin was the second highly used nonprescribed drug (11.9%), and Metronidazole was in the third position (10.6%). The following topical (local) antibiotics trimethoprim, gentamicin, and tetracycline were small in number in the nonprescribed medication scoring 1.8%, 1.3%, and 4.4%, respectively.

**Figure 2:**
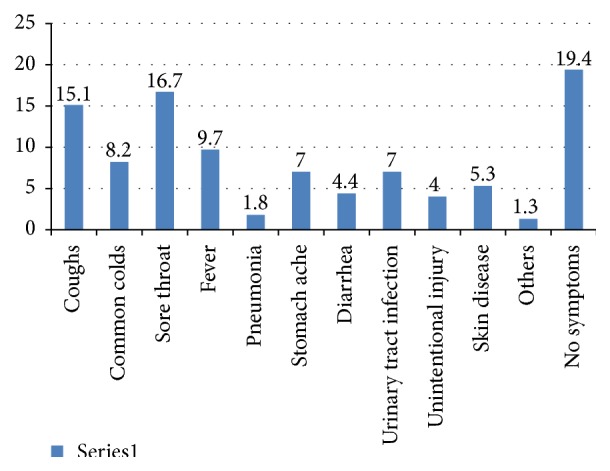
Disease for which antibiotics are self-prescribed (*n* = 536).

Participants have reported 10 different diseases for which antibiotics were self-prescribed. Respiratory diseases such as cough, sore throat, and common colds were reported as the most frequent illnesses for self-medication with antibiotics while other diseases such as diarrhea, urinary tract infections, and skin disease were among the least common illnesses for intended self-medication of antibiotics (Figure 2).

### 3.2. Multiple Logistic Regressions

Table 4 shows logistic regression for determinants of self-prescription of antibiotics using the backward stepwise method. We used three different methods (enter, forward stepwise, and backward stepwise) to determine which of the three would be the most suitable and parsimonious. After evaluation of these three methods, the best and the most parsimonious model would appear to be backward stepwise model as this model offers all significant factors which are common to all three models while retaining sufficiently high overall percentage which is correct.

We entered 17 variables into the model and evaluated the results. At each step we specified *p* = 0.05 for entry and *p* = 0.10 for removal from the model. *p* values in the table are final multivariate *p* values, which are obtained after adjustment for all other variables in the model. We found that nine factors were statistically significant in both univariate analysis and multiple logistic regression analysis indicating that they are not confounded by other factors; check Table 4.

## 4. Discussion

To our knowledge this study is the first of its kind examining predictors of self-medication with antibiotics in military hospitals in Riyadh City. Our results show that the prevalence of self-medication with antibiotics in Al Wazarat Health Center in Riyadh was 78.7%, which is very high. This result is certainly not unique to Saudi Arabia as it is comparable to several studies from the region and beyond which reported similar and even higher prevalence rates. In Eastern Province of Saudi Arabia, the prevalence of self-prescribed antibiotic was 80.0% [18]. In UAE study shows that the prevalence of self-prescribed antibiotics was 68.4%, while two studies from Sudan and Yemen have reported 79.5% and 78.0% prevalence rate of self-medication of antibiotics, respectively [19–21]. This high prevalence may not be surprising as most of antibiotics in Saudi Arabia and other countries in the region can be bought from private pharmacies without doctor's prescription.

This study found significant discrepancies in self-prescribed antibiotic among users. Among the respondents 70 or 13.0% were females, whereas 466 or 87.0% were males, although the number of female subjects in the present study was less than that of the male subjects; this finding is consistent with other studies [22]. The odds of being self-prescribed antibiotic user were higher among older respondents, as well as those with civil job type; this may indicate that military people are better well informed about the risks associated with self-medication or may be prudent to abide by the law and rules that prohibit the use and sale of medicines without prescription. In addition, respondents who are self-employed or retired had lower chances of being self-prescribed antibiotic user; this may be due to the fact that at older age people are more keen about their health and therefore they are supposed to visit doctors, so they are more likely to use prescribed antibiotics [13, 18, 23].

According to the study results, the most prevalent antibiotics used by study respondents were Amoxicillin, Ciprofloxacin, and Penicillin. These drugs are the common self-prescribed drugs and sometimes are used wrongly to treat common cold and other repertory infections and are also used wrongly as pain killer. Amoxicillin and Penicillin were reported to be the most common self-prescribed antibiotics in different countries [2, 24, 25]. The use of self-prescription may lead to the wrong use of antibiotics and inappropriate dosage which cause more harm than benefits [26–28]. Indications for antibiotic use revealed that self-prescribed antibiotics are mostly used for respiratory diseases such as sore throat, coughs, and common colds; this represents more than 50% of the overall symptoms reported by the study participants; this follows the pattern of other countries.

Finally the inappropriate use of antibiotics should not only be blamed on the patients alone as the healthcare providers and physicians in particular have a significant role to play by providing health education on self-prescription. Our study had serval limitations such as recall bias which was minimized by adapting a well formatted, simple, and easy-to-understand questionnaire. Multicenter study could not be carried out due to limited resources and as such Al Wazart Health Center was used. Al Wazart Health Center is one of the biggest primary health centers in Riyadh City which provides medical services for patients from all over the city. It attracts patients from all over the city due to its excellent location in the heart of the city and the availability of over twenty specialized clinics which are well equipped with both modern diagnostic equipment and highly qualified medical team. To ensure that the participants were representative of the target population the investigators used a two-stage cluster sampling method which can in fact be easily implemented and can create a more representative sample of the population.

## 5. Conclusion

This study revealed very high prevalence of self-prescribed antibiotics use which needs to be urgently addressed if the anticipated development of microbial resistance, cross-resistance, and treatment failure is to be avoided. The study also shows that Amoxicillin, Ciprofloxacin, and Penicillin were the most self-prescribed and misused antibiotics. Friend advice on antibiotics use and past experience of antibiotics used for treatment of similar illness were the main predictors for self-prescription with antibiotics.

## 6. Recommendations

Self-prescription with antibiotics is considered to be one of the most important issues affecting patients' treatment in hospitals around the country. The authors recommend intensive health education and promotion campaigns on the harm of self-prescription of antibiotics. The distribution of brochures to highlight the negatives of self-prescription and the correct use of antibiotics will go far in reducing self-prescription. Based on the study findings (see Table 3) the development of evidence-based guidelines for antibiotics use by the health authorities in Saudi Arabia is required. Urgent prospective studies to determine the level of microbial resistance and treatment failure among self-prescribed antibiotic users are highly recommended. Legislation by the Saudi government banning medication self-prescription will discourage and reduce such practice.

## Figures and Tables

**Table 1:** Distribution of calculated sample among the different clinics of Al Wazart Health Center using multistage cluster simple random sampling technique.

Selected clinics (first stage)	Patients seen between May and July 2014 in each clinic	Number of the randomly selected participants from each clinic (second stage)	Completed questionnaires for analysis
Chronic Disease Clinic (CDC)	3529	126	101
Dermatology Clinic	0800	126	120
ENT Clinic	0721	126	115
Flu Clinic	0815	126	124
Nephrology Clinic	0406	126	109
Neuro-Spinal Clinic	325	127	112
Total	**6596**	**757**	**681**

**Table 2:** Sociodemographic characteristics of participants and self-prescription of antibiotics (*n* = 681).

Variables	Self-prescribed antibiotics		Total (%)	OR (95% CI)
	No (%)	Yes (%)		
Gender				
Female	88 (55.7)	70 (44.3)	158 (23.2)	
Male	57 (10.9)	466 (89.1)	523 (76.8)	10.28 (6.771, 15.601)
Total	145 (21.3)	536 (78.7)	681 (100)	
Marital status				
Single	49 (24.0)	155 (76.0)	204 (30.0)	
Married	69 (22.0)	244 (78.0)	313 (46.0)	1.118 (0.736, 1.698)
Divorced	20 (16.3)	103 (83.7)	123 (18.1)	1.628 (0.915, 2.898)
Widow/widower	7 (17.1)	34 (82.9)	41 (6.0)	1.535 (0.640, 3.682)
Total	145 (21.3)	536 (78.7)	681 (100)	
Education				
No formal schooling	13 (13.0)	87 (87.0)	100 (14.7)	
Primary school	16 (8.6)	170 (91.4)	186 (27.3)	1.588 (0.731, 3.450)
Secondary school	13 (9.0)	132 (91.0)	145 (21.3)	1.517 (0.672, 3.428)
Diploma	53 (50.5)	52 (49.5)	105 (15.4)	0.147 (0.073, 0.294)
Degree	50 (34.5)	95 (65.5)	145 (21.3)	0.284 (0.144, 0.558)
Total	145 (21.3)	536 (78.7)	681 (100)	
Work status				
Not employed	28 (12.4)	198 (87.6)	226 (33.2)	
Employed	84 (24.1)	264 (75.9)	348 (51.1)	0.444 (0.279, 0.708)
Self-employed	21 (31.8)	45 (68.2)	66 (9.7)	0.303 (0.158, 0.582)
Retired	12 (29.3)	29 (70.7)	41 (6.0)	0.342 (0.157, 0.746)
Total	145 (21.3)	536 (78.7)	681 (100)	
Age				
18–30	56 (33.7)	110 (66.3)	166 (24.4)	
31–44	61 (21.3)	225 (78.7)	286 (42.0)	1.878 (1.223, 2.882)
≥45	28 (12.2)	201 (87.8)	229 (33.6)	3.655 (2.195, 6.084)
Total	145 (21.3)	536 (78.7)	681 (100)	
Job type				
Civil	49 (10.7)	409 (89.3)	458 (67.3)	
Military	96 (43.0)	127 (57.0)	223 (32.7)	0.158 (0.106, 0.236)
Total	145 (21.3)	536 (78.7)	681 (100)	

**Table 3:** Comparing the study findings of current study with similar studies.

Paper title	Study sitting	Study design	Sample size	Study duration	Prevalence of self-medicated antibiotics	Most common self-prescribed antibiotics
“Prevalence and Predictors of Self-Medication with Antibiotics in Al Wazarat Health Center, Riyadh City, KSA”	Al Wazarat Health Center, Riyadh, KSA	Cross-sectional study	757	February 2014 and November 2014	78.7%	Amoxicillin (22.3%), Ciprofloxacin (11.9%), and Metronidazole (10.6%)

“Self-Medication with Antibiotics in Rural Population in Greece: A Cross-Sectional Study” [29]	Southern Greece	Cross-sectional study	11139	Between November 2009 and January 2010	76.2%	Amoxicillin (18.3%), Amoxicillin/clavulanic acid (15.4%), and cefaclor (9.7%)

“Perceptions and Practices of Self-Medication among Medical Students in Coastal South India” [30]	South India	Cross-sectional study	440	March-April, 2011	78.6%	Antibiotics

“Self-Medication with Antibiotics for the Treatment of Menstrual Symptoms in Southwest Nigeria: A Cross-Sectional Study” [31]	Southwest Nigeria	Cross-sectional study	706	February 2008	24%	Ampicillin, tetracycline, Ciprofloxacin, and Metronidazole

“Prevalence of Self-Medication Practices and Its Associated Factors in Urban Puducherry, India” [32]	Puducherry, India	Cross-sectional study	352	December 2012-January 2013	11.9%	

“Self-Medication with Antibiotics in the Ambulatory Care Setting within the Euro-Mediterranean Region; Results from the ARMed Project” [33]	Cyprus, Egypt, Jordan, Lebanon, Libya, Tunisia, and Turkey	Cross-sectional study	2109	December 2007	19.1% in Cyprus, 37% in Lebanon, and 77% in Jordan	Antibiotics

“Patterns and Predictors of Self-Medication in Northern Uganda” [34]	Northern Uganda	Cross-sectional	884	November to December 2012	75.7%	Coartem (27.3%), amoxicillin (21.7%), Metronidazole (12.3%), and cotrimoxazole (11.6%)

**Table 4:** Multiple logistic regression (backward stepwise) of the use of “self-prescribed antibiotics” as dependent variable.

Independent variable	*β* (SE)	*p* value	Odds ratio (95% CI)
Friend advice (yes versus no)	1.999 (0.355)	0.000	7.382 (3.684, 14.791)
Pharmacy near (yes versus no)	1.277 (0.346)	0.000	3.584 (1.819, 7.061)
Psychology (yes versus no)	1.231 (0.355)	0.001	3.425 (1.707, 6.869)
Past experience (yes versus no)	2.435 (0.392)	0.002	11.413 (5.296, 24.594)
Mild illness (yes versus no)	1.057 (0.342)	0.002	2.879 (1.473, 5.626)
Gender (female versus male)	1.212 (0.356)	0.083	3.361 (1.673, 6.755)
Job type (working versus no working)	−1.104 (0.315)	0.087	0.332 (0.179, 0.615)
Age group ii (31–44)	0.449 (0.369)	0.223	1.567 (0.761, 3.229)
Age group iii (45 or more)	1.070 (0.416)	0.010	2.915 (1.289, 6.592)
Primary school	0.79 (0.571)	0.166	2.203 (0.720, 6.741)
Secondary school	0.886 (0.562)	0.115	2.426 (0.807, 7.293)
Diploma	−1.142 (0.527)	0.080	0.319 (0.114, 0.896)
No formal schooling	−0.814 (0.486)	0.094	0.443 (0.171, 1.150)

*p* values less than 0.05 are considered statistically significant.

## References

[1] Norris P. (2004). *Interventions to Improve Antimicrobial Use: Evidence from ICIUM 2004*.

[2] Togoobaatar G., Ikeda N., Ali M. (2010). Survey of non-prescribed use of antibiotics for children in an urban community in Mongolia. *Bulletin of the World Health Organization*.

[3] Cars O., Högberg L.

[4] Franchi C., Sequi M., Bonati M. (2011). Differences in outpatient antibiotic prescription in Italy's Lombardy region. *Infection*.

[5] World Health Organization (2001). *WHO Global Strategy for Containment of Antimicrobial Resistance*.

[6] Simonsen G. S., Tapsall J. W., Allegranzi B., Talbot E. A., Lazzari S. (2004). The antimicrobial resistance containment and surveillance approach—a public health tool. *Bulletin of the World Health Organization*.

[7] Tapsall J. W. (2005). Antibiotic resistance in *Neisseria gonorrhoeae*. *Clinical Infectious Diseases*.

[8] Stewart P. S., Costerton J. W. (2001). Antibiotic resistance of bacteria in biofilms. *The Lancet*.

[9] Gould I. M. (2009). Controversies in infection: infection control or antibiotic stewardship to control healthcare-acquired infection?. *Journal of Hospital Infection*.

[10] Balkhy H. H., Cunningham G., Chew F. K. (2006). Hospital- and community-acquired infections: a point prevalence and risk factors survey in a tertiary care center in Saudi Arabia. *International Journal of Infectious Diseases*.

[11] Pablos-Méndez A., Raviglione M. C., Laszlo A. (1998). Global Surveillance for Antituberculosis-Drug Resistance, 1994–1997. *New England Journal of Medicine*.

[12] Grigoryan L., Burgerhof J. G. M., Degener J. E. (2008). Determinants of self-medication with antibiotics in Europe: the impact of beliefs, country wealth and the healthcare system. *Journal of Antimicrobial Chemotherapy*.

[13] Garofalo L., Di Giuseppe G., Angelillo I. F. (2015). Self-medication practices among parents in Italy. *BioMed Research International*.

[14] Saradamma R. D., Higginbotham N., Nichter M. (2000). Social factors influencing the acquisition of antibiotics without prescription in Kerala State, south India. *Social Science & Medicine*.

[15] Esimone C. O., Nworu C. S., Udeogaranya O. P. (2007). Utilization of antimicrobial agents with and without prescription by out-patients in selected pharmacies in South-eastern Nigeria. *Pharmacy World & Science*.

[16] Zafar S. N., Syed R., Waqar S. (2008). Self-medication amongst university students of Karachi: prevalence, knowledge and attitudes. *Journal of the Pakistan Medical Association*.

[17] Field A. (2013). *Discovering Statistics Using IBM SPSS Statistics*.

[18] Khalil H., Abdullah W., Khawaja N. (2013). Self-prescribed antibiotics by Saudi patients as a routine self-management of dental problems. *Life Science Journal*.

[19] Shehnaz S. I., Agarwal A. K., Khan N. (2014). A systematic review of self-medication practices among adolescents. *Journal of Adolescent Health*.

[20] Belkina T., Al Warafi A., Hussein Eltom E., Tadjieva N., Kubena A., Vlcek J. (2014). Antibiotic use and knowledge in the community of Yemen, Saudi Arabia, and Uzbekistan. *Journal of Infection in Developing Countries*.

[21] Al Akhali K. M. (2013). Misuse of antibiotics and awareness of antibiotic hazard among the public and medical professionals in Thamar province, in Republic of Yemen. *Pharmacie Globale: International Journal of Comprehensive Pharmacy*.

[22] Llor C., Cots J. M. (2009). The sale of antibiotics without prescription in pharmacies in Catalonia, Spain. *Clinical Infectious Diseases*.

[23] Huttner B., Goossens H., Verheij T., Harbarth S. (2010). Characteristics and outcomes of public campaigns aimed at improving the use of antibiotics in outpatients in high-income countries. *The Lancet Infectious Diseases*.

[24] Nyazema N., Viberg N., Khoza S. (2007). Low sale of antibiotics without prescription: a cross-sectional study in Zimbabwean private pharmacies. *Journal of Antimicrobial Chemotherapy*.

[25] Viberg N., Kalala W., Mujinja P., Tomson G., Lundborg C. S. (2010). ‘Practical knowledge’ and perceptions of antibiotics and antibiotic resistance among drugsellers in Tanzanian private drugstores. *BMC Infectious Diseases*.

[26] Levy S. B. (1992). The antibiotic myth. *The Antibiotic Paradox*.

[27] Shah S. J., Ahmad H., Rehan R. B. (2014). Self-medication with antibiotics among non-medical university students of Karachi: a cross-sectional study. *BMC Pharmacology and Toxicology*.

[28] Afolabi A. (2012). *Self Medication, Drug Dependency and Self-Managed Health Care-A Review*.

[29] Skliros E., Merkouris P., Papazafiropoulou A. (2010). Self-medication with antibiotics in rural population in Greece: a cross-sectional multicenter study. *BMC Family Practice*.

[30] Kumar N., Kanchan T., Unnikrishnan B. (2013). Perceptions and practices of self-medication among medical students in coastal South India. *PLoS ONE*.

[31] Sapkota A. R., Coker M. E., Rosenberg Goldstein R. E. (2010). Self-medication with antibiotics for the treatment of menstrual symptoms in southwest Nigeria: a cross-sectional study. *BMC Public Health*.

[32] Selvaraj G., Kumar S. G., Ramalingam A. (2014). Prevalence of self-medication practices and its associated factors in Urban Puducherry, India. *Perspectives in Clinical Research*.

[33] Scicluna E. A., Borg M. A., Gür D. (2009). Self-medication with antibiotics in the ambulatory care setting within the Euro-Mediterranean region; results from the ARMed project. *Journal of Infection and Public Health*.

[34] Ocan M., Bwanga F., Bbosa G. S. (2014). Patterns and predictors of self-medication in northern Uganda. *PLoS ONE*.

